# Validation of a model to predict all-cause in-hospital mortality in vascular surgical patients

**Published:** 2008-11

**Authors:** BM Biccard, RR Pooran

**Affiliations:** Department of Anaesthetics, Nelson R Mandela School of Medicine and Inkosi Albert Luthuli Central Hospital, Durban, South Africa; Nuffield Department of Anaesthetics, University of Oxford, John Radcliffe Hospital, Oxford, UK; Inkosi Albert Luthuli Central Hospital, Durban

## Abstract

**Objective:**

To develop and validate a pre- and postoperative model of all-cause in-hospital mortality in South African vascular surgical patients.

**Methods:**

We carried out a retrospective cohort study. A multivariate analysis using binary logistic regression was conducted on a derivation cohort using clinical, physiological and surgical data. Interaction and colinearity between covariates were investigated. The models were validated using the Homer-Lemeshow goodness-of-fit test.

**Results:**

Independent predictors of in-hospital mortality in the pre-operative model were: (1) age (per one-year increase) [odds ratio (OR) 1.03, 95% confidence interval (CI) 1.0–1.06), (2) creatinine > 180 μmol.l^-1^ (OR 6.43, 95% CI: 3.482–11.86), (3) chronic beta-blocker therapy (OR 2.48, 95% CI: 1.38–4.48), and (4) absence of chronic statin therapy (OR 2.81, 95% CI: 1.15–6.83). Independent predictors of mortality in the postoperative model were: (1) age (per oneyear increase) (OR 1.05, 95% CI: 1.02–1.09), (2) creatinine > 180 μmol.l^-1^ (OR 5.08, 95% CI: 2.50–10.31), (3) surgery out of hours without statin therapy (OR 8.27, 95% CI: 3.36–20.38), (4) mean daily postoperative heart rate (HR) (OR 1.02, 95% CI: 1.0–1.04), (5) mean daily postoperative HR in the presence of a mean daily systolic blood pressure of less than 100 beats per minute or above 179 mmHg (OR 1.02, 95% CI: 1.01–1.03) and (6) mean daily postoperative HR associated with withdrawal of chronic beta-blockade (OR 1.02, 95% CI: 1.01–1.03). Both models were validated.

**Conclusion:**

The pre-operative model may predict the risk of in-hospital mortality associated with vascular surgery. The postoperative model may identify patients whose risk increases as a result of surgical or physiological factors.

## Summary

A model derived to estimate the risk of all-cause mortality following vascular surgery^1^ used cardiac risk factors,^2^ cardiac drug administration and the vascular surgical procedure performed. This is appropriate as cardiac complications account for approximately 60%^3^ to 75%^4^ of complications following vascular surgery. Similarly, the extent of the surgical procedure is associated with cardiac morbidity.^5^

In addition, it has previously been shown that simple physiological monitoring, including respiratory rate, heart rate (HR), systolic blood pressure and level of consciousness are important predictors of mortality in in-hospital patients.^6^ Physiological variables have been used in ‘early-warning scoring systems’ to identify patients at increased risk of mortality, requiring early intensive care unit admission.^7^

Integrating cardiac, surgical and physiological data into a model may allow identification of vascular surgical patients at risk of all-cause in-hospital mortality. We previously confirmed this in a study to determine independent predictors of all-cause in-hospital mortality following vascular surgery by analysing peri-operative physiological data, cardiac risk predictors and surgical procedural factors.^8^ This approach would allow a physician to identify high-risk patients pre-operatively, and then further stratify these patients in the postoperative period according to surgical factors and the physiological response to surgery. This may allow earlier identification and management of patients whose risk profile increases postoperatively, as a result of either surgical factors or the patient’s physiological response to the combination of surgery and pre-existing medical co-morbidities.

The aim of this study was to develop and validate a pre- and postoperative model of all-cause in-hospital mortality for South African vascular surgical patients.

## Methods

Local ethical approval for this study was granted by the ethics committee of the Nelson R Mandela School of Medicine. The patient cohort included all vascular surgical patients over 39 years of age, admitted for both elective and emergency vascular surgery at Inkosi Albert Luthuli Central Hospital (IALCH) between June 2003 and June 2007. The derivation cohort was defined as all patients who had had surgery between June 2003 and June 2006, and the validation cohort included all patients who had had surgery between July 2006 and June 2007. Only the last surgical procedure per patient was analysed.

From the hospital computerised database, we extracted demographic data associated with peri-operative cardiac risk,^2^ and physiological6 and surgical procedural data^5^,^9^ associated with in-hospital mortality. Data on the following clinical risk factors were collected: history of ischaemic heart disease (or pathological Q waves on ECG), history of congestive heart failure, diabetes, serum creatinine > 180 µmol.l^-1^, age, gender, pre- and postoperative blood haemoglobin and glucose levels, and a history of hypertension.

Factors obtained on vascular surgical procedures were type of surgery (supra-inguinal, carotid, infra-inguinal or other), duration of surgical cutting time, and whether surgery was undertaken out of elective surgical working hours.

The physiological data analysed included mean daily heart rate, systolic blood pressure (SBP) and diastolic blood pressure (DBP), which were obtained from nurses’ observations from the day prior to surgery until three days postoperatively. A further physiological variable was defined as the ‘last mean daily’ HR, SBP and DBP. These variables were defined as the mean daily HR, SBP and DBP, respectively, recorded on the third postoperative day. If the patient died or was discharged before the third postoperative day, the mean value for the day preceding death or discharge was used.

Chronic pre-operative beta-blocker and statin therapy was recorded. If any dose of beta-blocker was discontinued within the first three postoperative days, these patients were considered to have withdrawn from chronic beta-blockade in the peri-operative period. This information was obtained from the nursing drug administration charts.

The primary outcome analysed was in-hospital mortality. All patients who were discharged from hospital were considered to be survivors.

## Statistical analyses

All categorical data were analysed using descriptive statistics and either the Fisher’s exact test or Pearson’s chi-square test where appropriate. All continuous data were analysed using descriptive statistics and compared using the independent-samples *t*-test or Mann-Whitney U-test where appropriate. Using the derivation cohort, a pre- and postoperative model of in-hospital mortality was developed. For both models, bivariate identification of risk factors associated with in-hospital postoperative death was conducted using binary logistic regression analysis. Multivariate analysis was then conducted using binary logistic regression analysis with a backward stepwise selection technique based on likelihood ratios with entry and exit probabilities set to 0.05 and 0.1, respectively. Any variable with more than 10% of the data missing was excluded from the multivariate analysis.^10^

Interaction between independent predictors, and colinearity were also investigated. Colinearity was considered if Pearson’s correlation coefficient was > 0.6 or the standard error of a covariate was > 5.0.^11^ If colinearity was identified, the multivariate analysis was repeated after removal of the responsible covariate.^12^

The performance of the pre- and postoperative models was assessed by the Hosmer and Lemeshow goodness-of-fit test. A non-significant result would suggest that the validation model confirms the findings in the derivation cohort. A receiver operating characteristic curve (ROC) was constructed for both the pre- and postoperative models using the validation cohort. The odds ratios (OR) for all-cause mortality and 95% confidence intervals (CI) are reported. SPSS 15.0 for Windows (6 September 2006) was used for data analysis.

## Results

Eight hundred and twenty-nine vascular patients were identified in the whole cohort, of which 621 were in the derivation cohort and 198 in the validation cohort. Demographic, clinical, surgical and physiological data for both the derivation and the validation cohorts are presented in Table 1.

**Table 1:** Demographic, Cardiac Risk And Surgical Risk Factors And Postoperative Physiological Data For The Derivation And Validation Cohorts. Values Are Median (IQR) Or Number (Proportion)

*Characteristic*	*Derivation cohort (n = 631)*	*Validation cohort (n = 198)*	p*-value*
Pre-operative risk factors			
Age	62 (54–68)	61 (53–70)	0.429**
Gender (male)	423 (67)	136 (68.7)	0.728*
Diabetic	217 (34.4)	81 (40.9)	0.107*
Hypertensive	400 (63.5)	140 (70.1)	0.48*
Smoker	375 (59.5)	142 (72.4)	0.001*
Ischaemic heart disease	347 (55)	126 (64.3)	0.026*
Congestive cardiac failure	9 (1.4)	4 (2.1)	0.517*
Stroke	123 (19.5)	50 (25.4)	0.088*
Creatinine > 180 μmol.l^-1^	63 (10.5)	7 (4.1)	0.010*
Pre-operative glucose	5.9 (5.1–8.5)	5.9 (4–8)	0.627**
Pre-operative haemoglobin	12.7 (11.2–14.3)	12.9 (11–14)	0.457**
Pre-operative chronic medical therapy			
Statin therapy	103 (16.3 )	30 (15.2)	0.740*
Chronic beta-blockade	152 (24.1)	43 (21.7)	0.565*
Surgical risk factors			
Supra-inguinal surgery	80 (12.7%)	30 (15.2%)	0.401*
Duration of surgery (minutes)	103 (70-130)	113 (70-154)	0.203**
Surgery out of hours	52 (8.2%)	20 (10.1%)	0.249*
Postoperative physiological data and risk factors			
Withdrawal of chronic beta-blockade	96 (15.5%)	10 (5.1%)	< 0.001*
Mean last heart rate	83 (73–95)	79 (71–90)	0.002**
SBP < 100 or > 179 mmHg	52 (8.5)	10 (6.3)	0.362*
Mean last SBP	127 (112–141)	128 (117–145)	0.071**
Mean last DBP	81 (69–116)	79 (69–120)	0.965**
Postoperative glucose	7.9 (6.1–10)	7.8 (6.9–9.3)	0.627**
Postoperative haemoglobin	10.3 (8–11.8)	10.3 (9.3–11.5)	0.557**

*Fisher’s exact test; **Mann–Whitney test; SBP: systolic blood pressure; DBP: diastolic blood pressure; IQR: interquartile range.

The cohorts were similar, with the exception that the validation cohort had significantly more smokers and patients with ischaemic heart disease, and significantly fewer with an elevated serum creatinine or postoperative withdrawal of chronic beta-blockade. The patients in the validation cohort had a significantly lower postoperative heart rate than the derivation cohort.

The bivariate predictors of mortality are shown in Table 2. Although, pre-operative haemoglobin concentration and postoperative serum glucose were identified as bivariate predictors of mortality, neither of these variables were entered into the multivariate analysis, as more than 10% of these missing variables were absent from the derivation data set. Postoperative haemoglobin concentration and pre-operative serum glucose were also not entered into the multivariate analysis for the same reason. Therefore, out of 23 variables, 19 were subjected to multivariate analysis.

**Table 2:** Bivariate Analysis Of Predictors Of All-Cause Mortality Following Vascular Surgery In Patients ≥ 40 Years Of Age In The Derivation Cohort

*Characteristic*	*Odds ratio*	*95% CI*	p*-value*
Pre-operative risk factors			
Age (years)	1.037	1.010–1.064	0.006
Gender (male)	1.682	0.935–3.024	0.082
Diabetic	0.774	0.447–1.341	0.361
Hypertensive	0.962	0.570–1.625	0.885
Smoker	1.247	0.736–2.111	0.412
Ischaemic heart disease	1.041	0.627–1.728	0.876
Congestive cardiac failure	1.035	0.128–8.407	0.974
Stroke	0.519	0.242–1.117	0.094
Creatinine > 180 μmol.l^-1^	6.909	3.812–12.522	< 0.001
Pre-operative glucose	0.977	0.896–1.066	0.604
Pre-operative haemoglobin	0.881	0.778–0.997	0.044
Pre-operative chronic medical therapy			
Statin therapy	1.791	0.795–4.037	0.160
Chronic beta-blockade	2.147	1.267–3.639	0.589
Surgical risk factors			
Supra-inguinal surgery	0.991	0.590–1.663	0.971
Duration of surgery (minutes)	1.011	1.007–1.016	< 0.001
Surgery out of hours	3.176	1.599–6.309	0.001
Postoperative physiological data and risk factors			
Withdrawal of chronic beta-blockade	2.780	1.560–4.956	0.001
Mean last daily heart rate	1.021	1.005–1.038	0.012
Mean daily SBP < 100 or > 179 mmHg	4.006	2.026–7.920	0.000
Mean last daily SBP	1.002	0.990–1.014	0.767
Mean daily last DBP	1.006	0.981–1.031	0.637
Postoperative glucose	1.082	1.003–1.166	0.041
Postoperative haemoglobin	0.866	0737–1.018	0.081

CI: confidence interval; SBP: systolic blood pressure; DBP: diastolic blood pressure.

Seven independent predictors of in-hospital all-cause mortality were identified on multivariate analysis. These independent predictors are presented in Table 3. The pre-operative model was derived using pre-operative data alone and the postoperative model was derived using all the data available (Table 3). Multivariate analysis of the validation cohort alone identified no independent predictors for a pre-operative model. The independent predictors in the validation cohort of the postoperative model included age (OR 1.12, 95% CI: 1.01–1.24, *p* = 0.026) and last mean daily HR (OR 1.16, 95% CI: 1.08–1.26, *p* < 0.001).

**Table 3:** Multivariate Predictors Of Early Postoperative All-Cause In-Hospital Mortality Following Vascular Surgery In Patients ≥ 40 Years Of Age

	p*-value and odds ratio (95% CI)*			
	*Derivation model (n = 631)*		*Entire cohort (n = 829)*	
Pre-operative mortality model				
Age (per 1-year increase)	0.056	1.03 (1.0–1.06)	0.067	1.02 (1.0–1.05)
Creatinine > 180 μmol.l^-1^	< 0.001	6.43 (3.48–11.86)	< 0.001	5.36 (3.01– 9.55)
Chronic beta-blocker therapy	0.002	2.48 (1.38–4.48)	0.01	2.02 (1.18–3.46)
No chronic statin therapy	0.023	2.81 (1.15–6.83)	0.02	2.51 (1.12–5.64)
Postoperative mortality model				
Age (per 1-year increase)	0.001	1.05 (1.02–1.09)	0.001	1.05 (1.02–1.08)
Creatinine > 180 μmol.l^-1^	< 0.001	5.08 (2.50–10.31)	< 0.001	4.35 (2.24–8.42)
Surgery out of hours × no chronic statin therapy	< 0.001	8.27 (3.36–20.38)	< 0.001	5.31 (2.29–12.30)
Last mean daily HR (per beat per minute increase)	0.08	1.02 (1.0–1.04)	< 0.001	1.03 (1.02–1.05)
Last mean daily HR × ‘last mean daily SBP < 100 or > 179 mmHg’	< 0.001	1.02 (1.01–1.03)	< 0.001	1.02 (1.01–1.03)
Last mean daily HR × withdrawal of chronic beta-blockade	< 0.001	1.02 (1.01–1.03)	< 0.001	1.01 (1.01–1.02)

CI: confidence interval; HR: heart rate; SBP: systolic blood pressure.

No interaction between covariates or colinearity was detected when deriving the pre-operative model. The postoperative model revealed interaction between ‘duration of surgery’ and ‘last mean daily HR’, between ‘last mean daily HR’ and ‘mean daily SBP < 100 or > 179 mmHg’, between ‘surgery out of hours’ and ‘absence of chronic statin therapy’ and between ‘last mean daily HR’ and ‘withdrawal of chronic beta-blockade’. Colinearity was only identified between the covariates ‘last mean daily HR’, ‘duration of surgery’ and their interaction. The correlation coefficient for the two covariates was *r* = 0.890 and for each covariate with the interaction, it was *r* = –0.903 and *r* = –0.979, respectively. ‘Duration of surgery’ was removed for the model, and the subsequent multivariate analysis no longer revealed colinearity between the covariates. The model is presented in Table 3. The standard errors for all the covariates were between 0.003 and 0.46.

The goodness-of-fit for the pre- and postoperative models using the Hosmer and Lemeshow test is presented in Table 4. This test was insignificant for both the pre- and postoperative models. These results validate the pre- and postoperative mortality models. The optimal sensitivity and specificity of the pre-operative model were 83 and 40%, respectively. The optimal sensitivity and specificity of the postoperative model were 82 and 82%, respectively.

**Table 4:** Hosmer And Lemeshow Test And Area Under The ROC Curve For Pre- And Postoperative All-Cause In-Hospital Mortality In South African Vascular Surgical Patients

	*Percentage correct in cohort*		p*-value*	*Area under ROC curve (validation cohort)*
*Model*	*Derivation (%)*	*Validation (%)*		
Pre-operative model	88.2	91.2	0.291	0.583
Postoperative model	91.2	92.9	0.454	0.879

## Discussion

This study has validated a pre- and postoperative model of allcause in-hospital mortality in South African vascular surgical patients. It confirms the importance of clinical risk factors,^2^ medical therapy,^13^,^14^ surgical risk factors^3^,^5^ and physiological data^15^ in predicting in-hospital mortality.

The pre-operative model would allow for pre-operative prediction of mortality. This could be considered the baseline risk of the patient. The low specificity of the pre-operative model in comparison with the postoperative one illustrates how important surgical and physiological haemodynamic factors are for postoperative survival. Use of the postoperative model following surgery would allow for correction of subsequent surgical factors and the patient’s physiological response to surgery. Identification of increasing risk of mortality in the postoperative period may allow for earlier appropriate investigation, referral to high-dependency units and other therapeutic interventions, which may improve outcome.^15^ This postoperative model may therefore function as an early-warning system.

Postoperative physiological data obtained from routine nursing observations has been shown in this study to have prognostic importance, independent of cardiac clinical risk predictors for survival following vascular surgery. Attention to routine postoperative nursing observations may be sufficient to identify patients at increasing risk of mortality. This is particularly relevant in the postoperative ward following intensive care or high-dependency unit discharge, where intensive monitoring (such as ECG or invasive arterial blood pressure monitoring) and special investigations (troponin sampling) may not be routine.

This study confirms the importance of chronic medical therapy.^16^ Withdrawal of chronic beta-blockade was associated with mortality, as has been previously reported.^16^ A history of chronic beta-blockade is a risk factor in the pre-operative model, as these patients may potentially be withdrawn from therapy in the postoperative period. This is the first study to identify that the omission of a single dose of beta-blocker therapy within the first three postoperative days is an independent predictor of mortality. Previously, studies have considered withdrawal throughout the postoperative period.^13^ Clearly, even short periods of withdrawal postoperatively are important (Table 3).

It is possible that an awareness of the importance of withdrawal of chronic beta-blockade at our institution has resulted in the significant reduction in this event in our validation cohort (Table 1). Previously, it has been suggested that the beta-receptor upregulation associated with chronic beta-blockade may be responsible for the mortality associated with chronic beta-blockade in the peri-operative period.^16^ Indeed, our article is also the first to show that the mortality associated with withdrawal of chronic beta-blockade is associated with an increasing mean daily heart rate at the time of withdrawal, which is consistent with the potential morbidity associated with beta-receptor upregulation.

In our models, chronic pre-operative statin therapy was shown to be associated with a survival benefit for all vascular surgery in the pre-operative model, and importantly for out-ofhours surgery in the postoperative model. This is surprising, as the current evidence is only suggestive of peri-operative cardioprotective efficacy associated with statin therapy.^17^ In the light of this observation, it is possible that the survival benefit associated with statin therapy in this study may not necessarily be entirely attributable to statin therapy, but it may also be a surrogate marker of access to healthcare and long-term medical therapy prior to admission for vascular surgery. It may be this additional factor associated with chronic pre-operative statin therapy, and not statin therapy *per se* that could be associated with survival (Table 3).

The colinearity identified between the duration of surgery and the mean postoperative HR may partly explain why manipulation of postoperative HR with acute peri-operative beta-blockade alone is not necessarily cardioprotective.^18^ Clearly, the relationship between the extent of the surgical procedure and the associated physiological response has a profound effect on postoperative HR and subsequent survival.

It was hoped that the findings in this study would allow for continued prognostication for our patients throughout the perioperative period and hospital stay. Traditionally, anaesthetists have become accustomed to risk indices that are presented as prognostic tables. By convention, the patients are graded into classes.^2^,^19^ The scoring for each class is derived from either the summation of variably weighted risk factors, determined by the proportional weighting of the independent risk factors (such as Goldman’s risk index)^19^ or by summation of equally weighted variables (such as Lee’s revised cardiac risk index).^2^ These prognostic scores are, however, predominantly reliant on dichotomous data, with either the presence or absence of a clinical risk factor.

Integration of physiological data (such as age and heart rate) introduces continuous data into the model. Due to the exponential relationship associated with continuous data in logistic regression models, these models are not easily converted to prognostic tables. Indeed, they are rather cumbersome, as they require a number of weighted scores for each continuous variable. For this reason, it is suggested that a spreadsheet be constructed for both the pre- and postoperative models to calculate mortality risk. This is fairly easy using the logistic regression equation:

*p* = 1/ [1 + *e* –*^(b0 + b_1_ x_1_ + b_2_ x_2_ +… b_k_ x_k_)^*]

where *p* is probability of mortality, *e* is the exponent, *b*_0_ is the coefficient of the constant, *b*_1_ to *b*_k_ are the coefficients of the independent predictors, and *x*_1_ to *x*_k_ are values of the independent predictors.

The coefficients of the necessary constants and independent predictors derived in these models are reported in Table 5. For dichotomous covariates, the absence of the covariate is represented by 0 and its presence by 1 in the logistic regression equation.

**Table 5:** Coefficients Of The Constant And Independent Predictors For The Pre- And Postoperative Mortality Models

*Model and independent predictor*	*Coefficients*
Pre-operative model	
Constant	–5.269
Age (per one-year increase)	0.027
Creatinine > 180 μmol.l^-1^	1.86
No chronic beta-blocker therapy	0.91
No chronic statin therapy	1.032
Postoperative model	
Constant	–7.951
Age (per one-year increase)	0.052
Creatinine > 180 μmol.l^-1^	1.625
Surgery out of hours × no chronic statin therapy	2.113
Last mean daily HR (per beat per minute increase)	0.017
Last mean daily HR × ‘last mean daily SBP < 100 or > 179 mmHg’	0.019
Last mean daily HR × withdrawal of chronic beta-blockade	0.017

Although a spreadsheet is recommended, as the pre-operative model has only a single continuous variable (age), a pre-operative nomogram is presented in Table 6 which has been derived from the entire cohort. This is a variable-weighted risk index, where the weight per variable was determined by multiplying the corresponding OR by 10 in order to calculate whole numbers for the nomogram. Four classes are defined, with transition between classes defined by the 25th, 50th and 75th percentiles. This could be reproduced as a card in the pre-operative clinic.

**Table 6:** A Pre-Operative Nomogram: Risk Calculator

*Risk factor*	*Definition of variable*	*Points*
Age (years)	40	10
	41–45	11
	46–50	13
	51–55	15
	56–60	17
	61–65	20
	66–70	22
	71–75	26
	76–80	29
	81–85	34
	86–90	39
	> 90	44
Serum creatinine (μmol.l^-1^)	< 180	10
	> 180	54
Chronic pre-operative beta-blockade	No	10
	Yes	20
Chronic pre-operative statin therapy	No	25
	Yes	10
Pre-operative score = sum of age, creatinine, beta-blocker and statin therapy		
Class*	*Score definition*	*Mortality (%)*
Class 1	≤ 60	4
Class 2	61–65	8
Class 3	66–72	10
Class 4	> 72	23

*Pearson chi-square, *p* < 0.001.

It is important to appreciate that as only the last procedure per patient was analysed, the predicted mortality in these models should be considered as a predicted mortality per patient admission, as opposed to per procedure. To demonstrate this point and the utility of these models, it is helpful to consider the following example. A 62-year-old male patient on statin therapy presents for elective vascular surgery. Pre-operatively, he has a predicted mortality of 2.7%. Elective surgery is carried out, and postoperatively he has a mean daily HR of 80 bpm and a SBP between 100 and 180 mmHg. He now has a predicted postoperative mortality of 3.3%. On the third postoperative day, his mean HR climbs to 100 bpm and his predicted mortality to 4.6%. He subsequently undergoes emergency surgery for a threatened limb. Postoperatively, his HR remains at 100 bpm and his SBP falls to 90 mmHg. His predicted mortality now rises to 23.9%.

There are limitations to this study. This was a retrospective study that relied on administrative data and medical records, based on physician records of important co-morbidities. It was therefore not possible to identify all clinical risk factors. It is likely that other clinical risk factors will be found to be important predictors of mortality and will need to be included in subsequent models.

The following shortcomings of this article are highlighted and should be considered in future research to improve the prognostic performance of the models presented in this article. Firstly, important physiological data such as pre-operative haemoglobin concentration and postoperative glucose levels were not included in the multivariate analysis, due to more than 10% of the data being missing. These two variables require additional investigation in future studies, as it is possible that they may have been independent predictors of outcome in our patients.

Secondly, as we could identify only the last surgical procedure in this study, we could not evaluate the importance of multiple vascular surgical procedures on in-hospital mortality. It is likely that this may be an important factor that should be studied in future when developing integrated risk indices, such as the models presented in this article.

Finally, thoracic epidural analgesia has been associated with improved cardiac outcomes in aortic vascular surgery.^20^ Conversely, morphine administration has been associated with increased mortality in non-ST-segment elevation acute coronary syndromes in medical (non-surgical) patients.^21^ Although, the mode and adequacy of analgesia was not evaluated in this study, it is likely to have important effects on both outcome and physiological variables.

## Conclusion

A simple pre- and postoperative model is presented, which may assist in prediction of in-hospital mortality in South African vascular surgical patients. It may be used as an early-warning system, should the predicted mortality increase during the hospital stay, which may allow for earlier investigation and intervention in an attempt to improve patient outcome. This model is easy to integrate into clinical practice using a dedicated spreadsheet.
